# A supplement, not a substitute: Accuracy and completeness of ChatGPT responses for common elbow pathology

**DOI:** 10.1177/17585732251365178

**Published:** 2025-10-06

**Authors:** Benjamin Fiedler, Umar Ghilzai, Abdullah Ghali, Phillip Goldman, Pablo Coello, Michael B. Gottschalk, Eric R Wagner, Adil Shahzad Ahmed

**Affiliations:** 1 Joseph Barnhart Department of Orthopedic Surgery, 3989 Baylor College of Medicine, Houston, TX, USA; 2 Department of Orthopedic Surgery, 1371 Emory University, Atlanta, GA, USA

**Keywords:** epicondylitis, cubital tunnel, distal biceps rupture, elbow arthritis, chatGPT, large language model

## Abstract

**Hypothesis:**

Large language models (LLMs) like ChatGPT have increasingly been used as online resources for patients with orthopedic conditions. Yet there is a paucity of information assessing the ability of LLMs to accurately and completely answer patient questions. The present study comparatively assessed both ChatGPT 3.5 and GPT-4 responses to frequently asked questions on common elbow pathologies, scoring for accuracy and completeness. It was hypothesized that ChatGPT 3.5 and GPT-4 would demonstrate high levels of accuracy for the specific query asked, but some responses would lack completeness, and GPT-4 would yield more accurate and complete responses than ChatGPT 3.5.

**Methods:**

ChatGPT was queried to identify five most common elbow pathologies (lateral epicondylitis, medial epicondylitis, cubital tunnel syndrome, distal biceps rupture, elbow arthritis). ChatGPT was then queried on the five most frequently asked questions for each elbow pathology. These 25 total questions were then individually asked of ChatGPT 3.5 and GPT-4. Responses were recorded and scored on 6-point Likert scale for accuracy and 3-point Likert scale for completeness by three fellowship-trained upper extremity orthopedic surgeons. ChatGPT 3.5 and GPT-4 responses were compared for each pathology using two-tailed *t*-tests.

**Results:**

Average accuracy scores for ChatGPT 3.5 ranged from 4.80 to 4.87. Average GPT-4 accuracy scores ranged from 4.80 to 5.13. Average completeness scores for ChatGPT 3.5 ranged from 2.13 to 2.47, and average completeness scores for GPT-4 ranged from 2.47 to 2.80. Total average accuracy for ChatGPT 3.5 was 4.83, and total average accuracy for GPT-4 was 5.0 (*p* = 0.05). Total average completeness for ChatGPT 3.5 was 2.35, and total average completeness for GPT-4 was 2.66 (*p* = 0.01).

**Conclusion:**

ChatGPT 3.5 and GPT-4 are capable of providing accurate and complete responses to frequently asked patient questions, with GPT-4 providing superior responses. Large language models like ChatGPT have potential to serve as a reliable online resource for patients with elbow conditions.

## Introduction

The advent of the Internet and ubiquitous access has ushered increased utilization and reliance upon online tools for health information. Resources such as WebMD, Wikipedia, Google, and Youtube are frequently utilized by patients to glean insight into their ailments.^1–3^ However, information quality via online resources is highly variable. While some information may be reliable and appropriately guide patient decision-making, other sources provide inaccurate information that dangerously misleads patients.^1,4,5^

In recent years, artificial intelligence (AI) systems have emerged as new and increasingly used resources for patients seeking health information. Via machine learning, AI platforms employ software algorithms to identify patterns in large datasets, creating increasingly accurate predictions.^6^ Large language models (LLMs) are a subset utilizing machine learning to both produce and comprehend human-like text.^7^ While multiple LLMs currently exist, one of the most popular is Chat-Generative Pre-Trained Transformer (ChatGPT, OpenAI, San Francisco, California). With over 100 million monthly users and 10 million daily queries,^8^ ChatGPT is one of the most visited websites in the world.

Application of LLMs like ChatGPT is already occurring in medicine, and even specifically in Orthopaedic Surgery. Recent versions of ChatGPT have passed the United States Medical Licensing Exam^9^ and further performed at the level of the average intern on the Orthopaedic In-Training Exam.^10^ It has even exhibited competence and ability to pass surgical subspecialty examinations, such as the American Shoulder and Elbow Surgeons (ASES) maintenance of certification exam (MOC).^11^ However, there is some concern regarding the accuracy and completeness of ChatGPT's responses, which is particularly relevant for patient-directed inquiries. Early research suggests ChatGPT may provide accurate and complete responses to simulated patient medical questions; however, there is variability in appropriate responses to different types of questions.^12^ Furthermore, accuracy and completeness of ChatGPT responses have not been assessed for specific orthopedic conditions, such as common elbow pathology.

To better assess ChatGPT as an appropriate resource for patients with elbow pathology, the present study evaluated the quality of sequential generations of ChatGPT (ChatGPT 3.5 and GPT-4) for responses to five common elbow conditions: lateral epicondylitis, medial epicondylitis, cubital tunnel syndrome, elbow arthritis, and distal biceps tendon rupture. All responses were graded for accuracy and completeness. We hypothesized that ChatGPT would demonstrate high levels of accuracy for the specific query, but some responses would lack completeness (Table 1).

**Table 1.** Average accuracy rating across responses of chat GPT 3.5 vs GPT-4 including *p*-values.

Condition	Average accuracy rating chat GPT-3.5	Average accuracy rating GPT-4	*p*-Value
Lateral epicondylitis	4.8	4.8	1.0
Medial epicondylitis	4.87	4.93	0.94
Cubital tunnel syndrome	4.87	5.13	0.75
Distal biceps rupture	4.8	5.07	0.76
Elbow arthritis	4.8	5.07	0.73

## Methods

ChatGPT 3.5 was queried to identify the five most common elbow conditions for which patients present to orthopedic surgeons. ChatGPT 3.5 was then asked to identify the five most frequently asked questions for each condition. These 25 total questions were then separately asked to both ChatGPT 3.5 and GPT-4 and responses recorded. The 25 questions and corresponding responses for both versions were then independently reviewed by three fellowship-trained upper extremity surgeons. Quality response was evaluated on 6-point Likert scale for accuracy and 3-point Likert scale for completeness. For completeness, a score of 1 indicated incomplete response, 2 indicated adequate response with minimum information required to be considered complete, and 3 indicated comprehensive response beyond what was expected. For accuracy, a score of 1 indicated completely incorrect, 2 indicated more incorrect than correct, 3 indicated equal parts correct and incorrect, 4 indicated more correct than not, 5 indicated nearly all correct, and 6 indicated a completely correct answer.

Average accuracy and completeness scores for each of the five questions for each elbow pathology were calculated and recorded. Statistical analysis using two-tailed *t*-tests was performed to assess for statistically significant differences in accuracy or completeness between ChatGPT versions 3.5 and GPT-4. Inter-rater reliability was assessed via intraclass coefficient (Table 2).

**Table 2.** Average completeness rating across responses of chat GPT 3.5 vs chat GPT 4.0 including *p*-values.

Condition	Average completness rating chat GPT-3.5	Average completeness rating GPT-4	*p*-Value
Lateral epicondylitis	2.47	2.47	0.17
Medial epicondylitis	2.47	2.67	0.47
Cubital tunnel syndrome	2.27	2.8	0.47
Distal biceps rupture	2.13	2.67	0.47
Elbow arthritis	2.4	2.67	1

## Results

**Figure 1.**
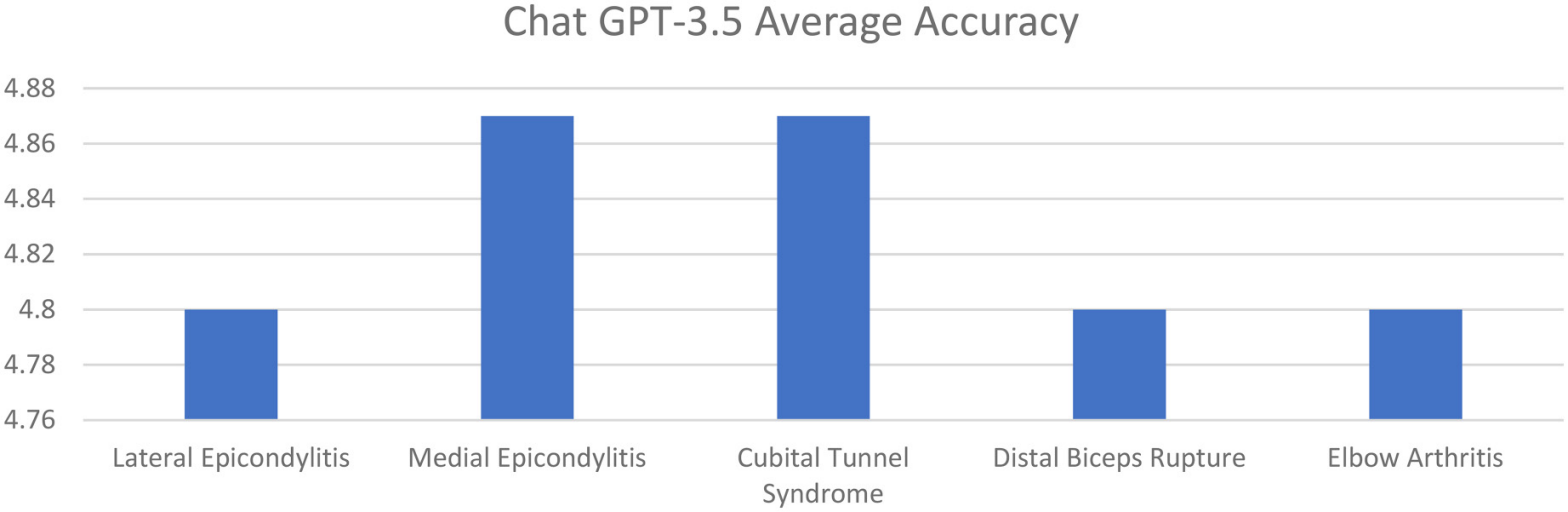
Average response accuracy of chat GPT 3.5 for elbow pathologies (0–6 point scale).

**Figure 2.**
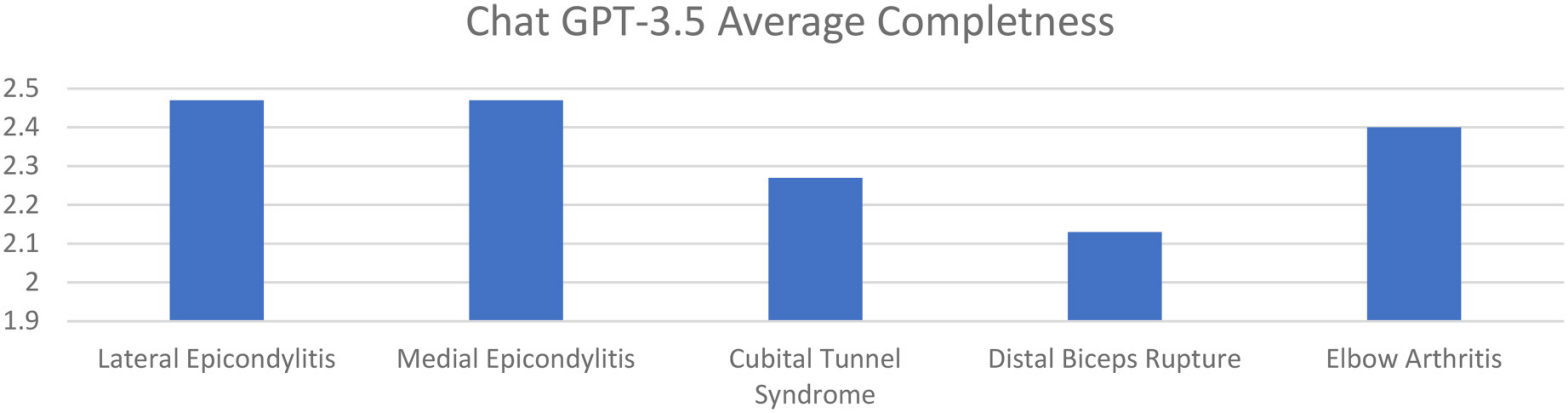
Average completeness of responses of chat GPT 3.5 for elbow pathologies (0–3 point scale).

Average accuracy scores for ChatGPT 3.5 for lateral epicondylitis, medial epicondylitis, cubital tunnel syndrome, distal biceps tendon rupture, and elbow arthritis were 4.80. 4.87, 4.87, 4.80, and 4.80, respectively. Average completeness scores for ChatGPT 3.5 for lateral epicondylitis, medial epicondylitis, cubital tunnel syndrome, distal biceps tendon rupture, and elbow arthritis were 2.47, 2.47, 2.27, 2.13, and 2.40, respectively (Figures 1 and 2).

**Figure 3.**
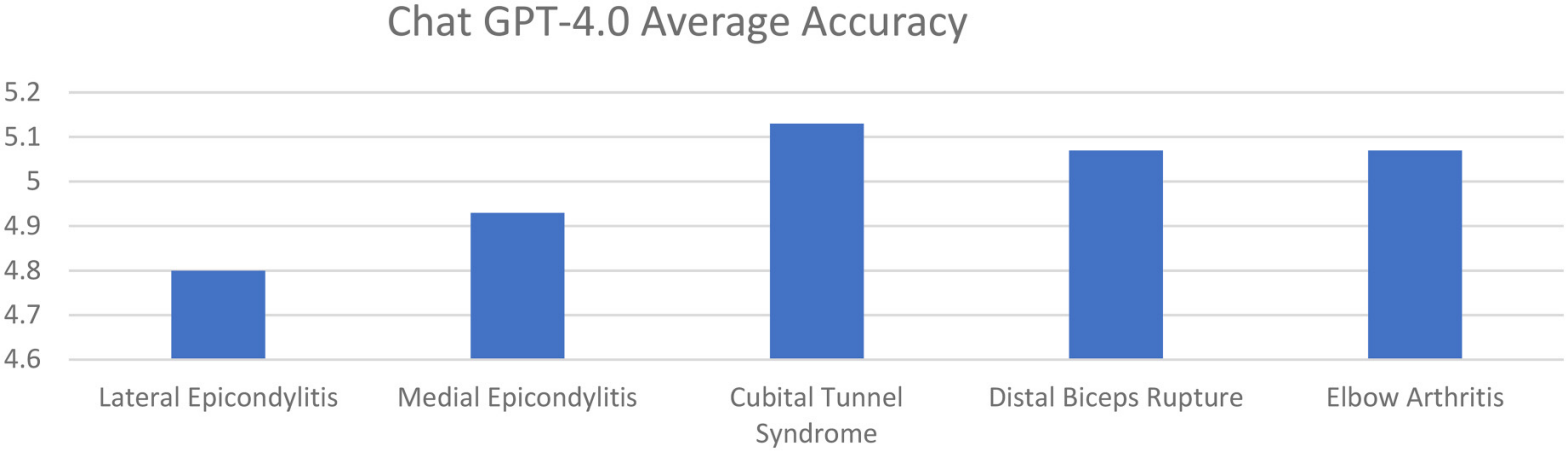
Average response accuracy of GPT-4 for elbow pathologies (0–6 point scale).

**Figure 4.**
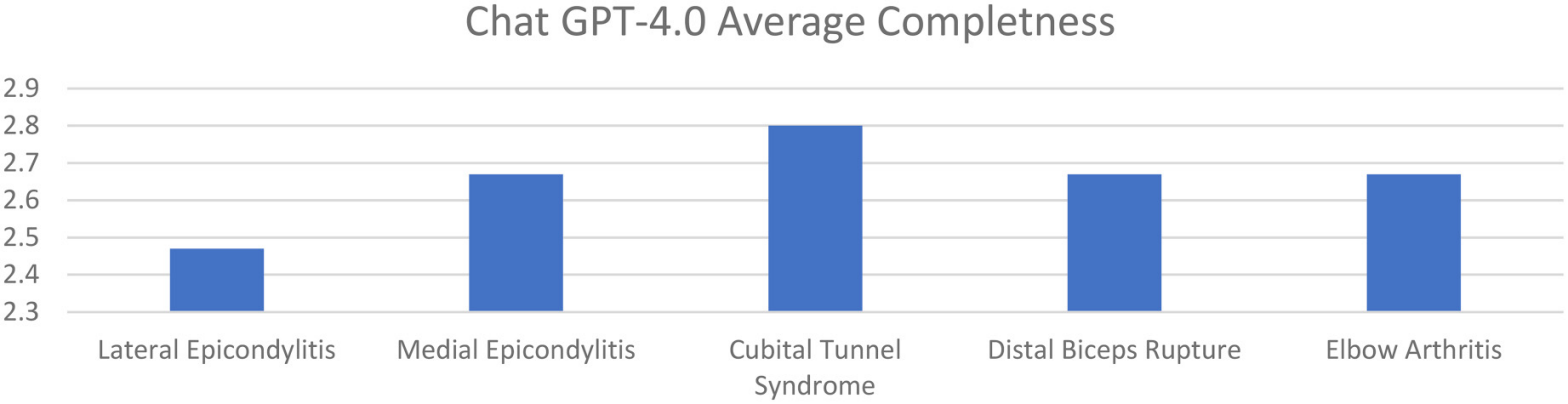
Average completeness of responses of GPT-4 for elbow pathologies (0–3 point scale).

Average accuracy scores for GPT-4 for lateral epicondylitis, medial epicondylitis, cubital tunnel syndrome, distal biceps tendon rupture, and elbow arthritis were 4.80, 4.93, 5.13, 5.07, and 5.07, respectively. Average completeness scores for GPT-4 for lateral epicondylitis, medial epicondylitis, cubital tunnel syndrome, distal biceps tendon rupture, and elbow arthritis were 2.47, 2.67, 2.80, 2.67, and 2.67, respectively. There was no statistically significant difference in accuracy or completeness of response for any individual elbow pathology between ChatGPT 3.5 and GPT-4. However, there were significant differences favoring GPT-4 when comparing overall averages for both accuracy and completeness (Figures 3 and 4) (Table 3).

**Table 3.** Average overall accuracy and completeness comparison between ChatGPT 3.5 and GPT-4.

	ChatGPT 3.5	GPT-4	*p*-Value
Average accuracy	4.83	5	0.05
Average completeness	2.35	2.66	0.01

**Figure 5.**
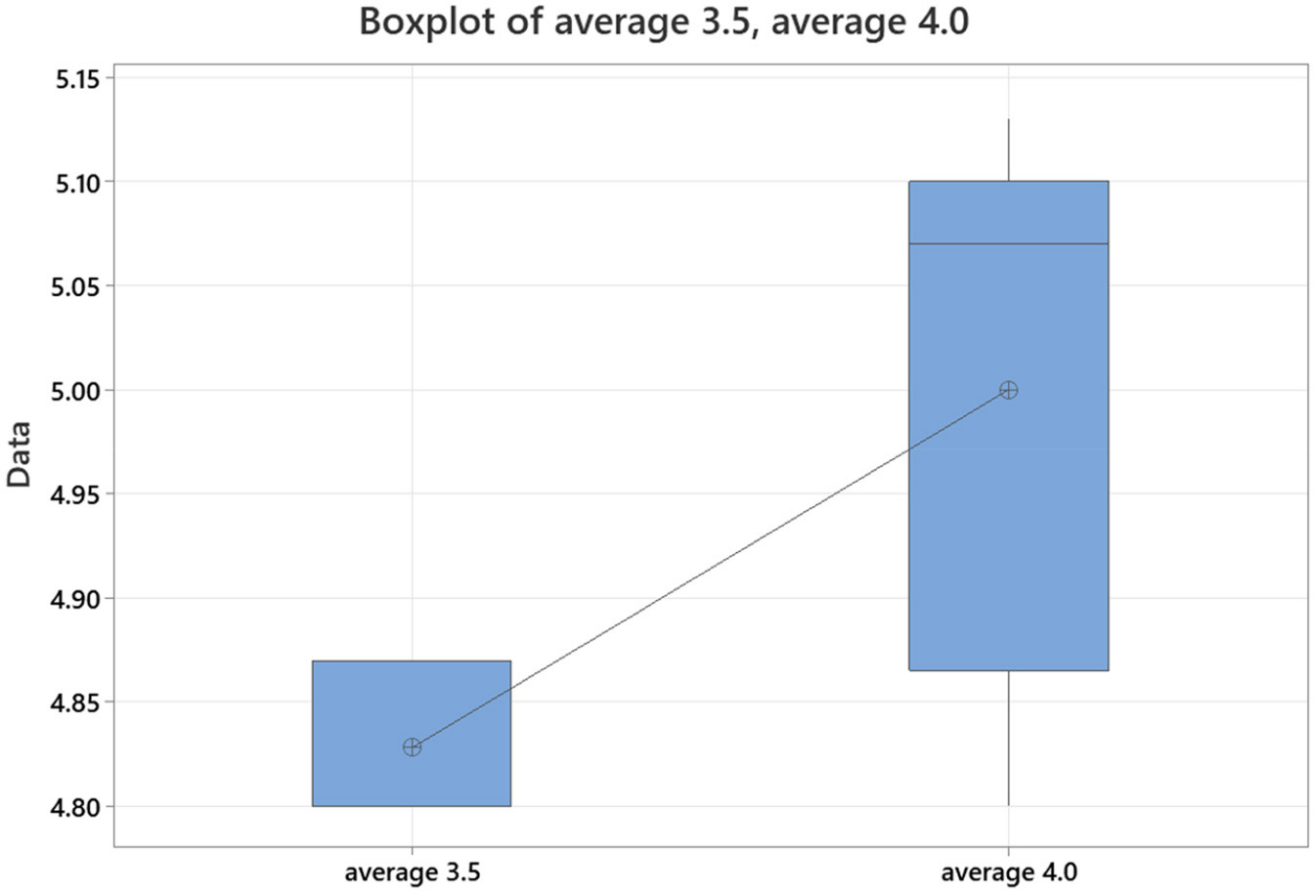
Comparison of average response accuracy for ChatGPT-3.5 vs GPT-4.

**Figure 6.**
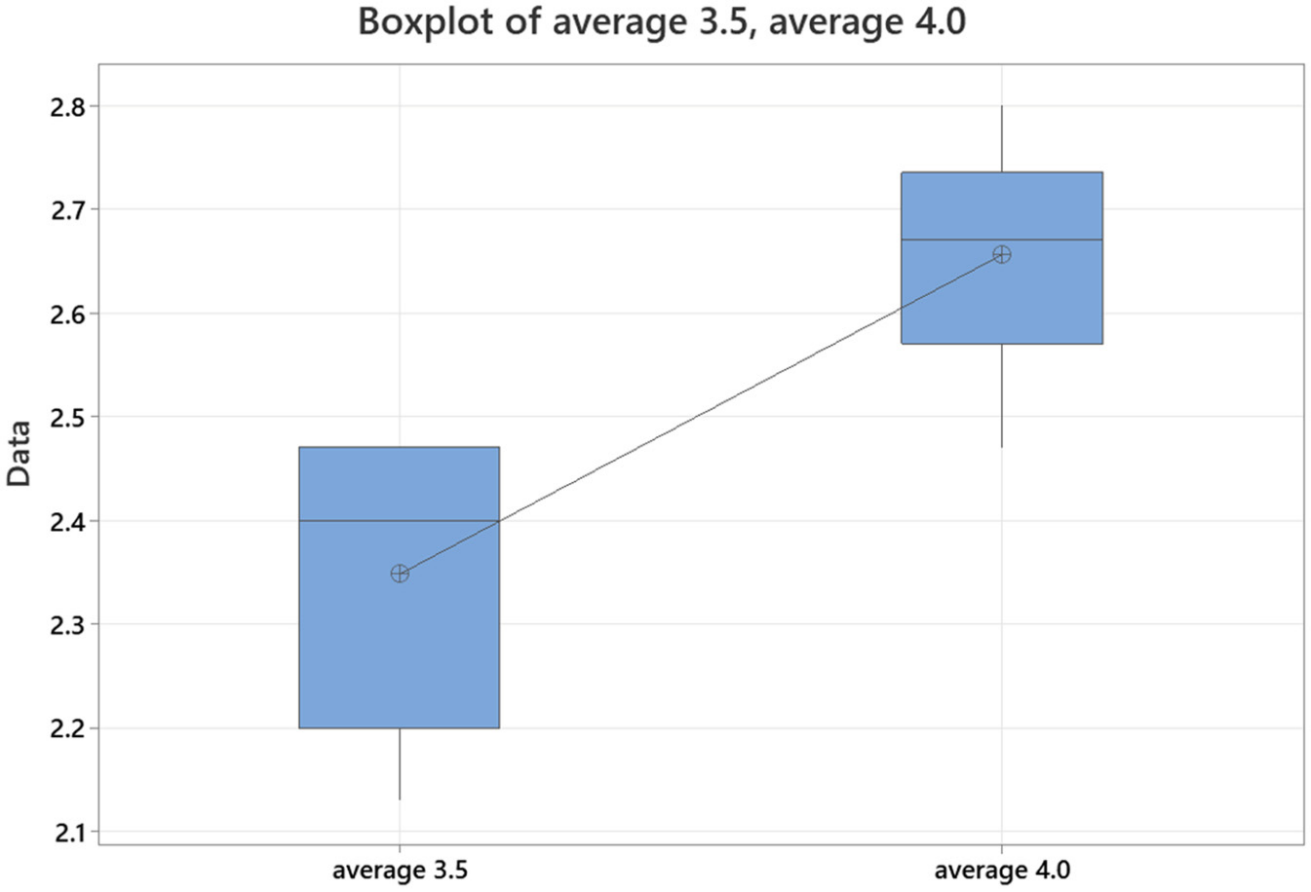
Comparison of average response completeness for chat GPT-3.5 vs GPT-4.

The average intraclass coefficient for completeness scoring in ChatGPT 3.5 was 0.93 (0.70–0.99). Average intraclass coefficient for accuracy scoring in ChatGPT 3.5 was 0.77 (0.01–0.97). The average intraclass coefficient for completeness scoring in GPT-4 was 0.97 (0.86–1.00). Average intraclass coefficient for accuracy scoring for GPT-4 was 0.72 (0.20–0.96). When comparing scoring for completeness and accuracy between ChatGPT 3.5 and GPT-4 across all responses, the correlation coefficient for completeness scoring was 0.96 (<0.001) and for accuracy scoring was 0.92 (<0.005) (Figures 5 and 6).

## Discussion

The present study evaluated ChatGPT's capacity to provide complete and accurate responses to patient inquiries on common elbow pathology. The results demonstrate that both versions ChatGPT 3.5 and GPT-4 produce responses that are accurate and moderately complete. The newer GPT-4 significantly outperformed ChatGPT 3.5 when comparing overall response accuracy and completeness.

While peer-reviewed literature and medical textbooks certainly offer reliable information, they are challenging and uncommon tools for patients to reliably access and understand. Subsequently, patients more and more often turn to online sources for readily available and easy-to-digest information. Discerning the veracity of information from the plethora of online tools can be challenging. Results from the present study suggest that ChatGPT may be a valuable tool for patients with elbow pathology. Of particular interest is the apparent capability of LLMs like ChatGPT to improve across newer generations of the algorithm. The overall scores for both accuracy and completeness were significantly better for GPT-4 compared to ChatGPT 3.5. However, these LLMs still have room to improve, especially regarding the completeness of responses. Needless to say, the ChatGPT interface is by no means a replacement for physician communication.

Utility of ChatGPT as a patient aid has recently been evaluated across various orthopedic specialities including joints, sports, spine, foot and ankle, shoulder surgery, and hand surgery.^13–16^ Results from studies in the aforementioned fields show that ChatGPT provides accurate, but less complete answers to the majority of patient questions. However, virtually all of these studies assess only a single generation of ChatGPT and do not assess for improvement across generations. The present study demonstrated a similar pattern of accurate but less complete responses, and the data further illustrated improved performance across newer versions. While there is substantial room for chatbot improvement, these findings highlight a progression across GPT generations to serve as an evolving patient education tool.

Findings from the present study provide additional context to the growing body of evidence demonstrating the utility of LLMs as medical information tools. A study comparing performance of ChatGPT-3.5 and GPT-4 against orthopedic residents on simulated boards-style questions found that residents scored highest at 74.2%, followed by GPT-4 at 47.2% and ChatGPT-3.5 at 29.4%. Similar to the present study, GPT-4 performed better than ChatGPT-3.5 on questions designed for subspecialized surgical trainees. Interestingly, when questions were categorized into text-only and image-inclusive, both ChatGPT versions performed better on text-only questions. ChatGPT has also been tested on capacity to understand and answer subspecialty-specific questions designed for fellowship-trained orthopedic surgeons. When tested on the ASES MOC, GPT-4 answered 60.2% of questions correctly, but was significantly inferior to shoulder and elbow fellowship-trained surgeons, who scored 75.3% correctly.^11^ Although these highlight continued improvement across generations of the LLM, they still fail to perform at the same level as surgeons on assessment of subspecialty knowledge.

Despite promising utility, employing ChatGPT in healthcare settings presents various issues. Multiple studies have reported on the “hallucination” phenomenon, in which ChatGPT provides fabricated information disguised as fact.^17,18^ This is especially common with scientific article citations, appropriately raising concerns regarding misinformation, patient safety, and scientific integrity when using ChatGPT in the healthcare setting. The propensity for this hallucination phenomenon, combined with the challenge of identifying this for patients, reinforces the importance that patients verify information from ChatGPT and other LLMs with their physician.^17,18^

Ultimately, ChatGPT provides useful and accurate information regarding elbow pathology. However, the lack of completeness in response and possibility of providing “hallucinated information” highlight potential areas of improvement in LLMs like ChatGPT. While the findings of this study show promise, it's vital that patients view LLMs as a supplement to their physician visit rather than a substitute.

## Limitations

The present study is not without limitations. The Likert-style questions, though common in research, reflect opinions and can be subject to bias. Further studies employing more objective scoring and assessment methods for LLM responses to patient queries would be beneficial. Additionally, this study focused on questions about common elbow pathology. These data cannot be generalized to ChatGPT's performance with less common pathology or complex questions. Moreover, this study only assessed ChatGPT version 3.5 and GPT-4. No other LLMs were analyzed in the present study, and assessment of additional LLM responses to elbow pathology questions may provide useful context, as the training datasets and pattern recognition between proprietary software may vary. Lastly, three fellowship-trained upper extremity surgeons independently assessed the LLM's responses in the present study. Utilizing additional orthopedic surgeons to assess response quality may provide more comprehensive data.

## Conclusion

ChatGPT overall provides accurate and comprehensive information on common elbow pathology, with improvement across newer generations of the LLM. While ChatGPT appears fairly reliable, several factors urge caution in its stand-alone effectiveness for patient education. Surgeons should remain aware of the types of information patients may glean from ChatGPT and similar LLMs, and possibly more important—the limitations and potential for misinformation amongst these LLMs.
